# Artificial cerebellum on FPGA: realistic real-time cerebellar spiking neural network model capable of real-world adaptive motor control

**DOI:** 10.3389/fnins.2024.1220908

**Published:** 2024-04-25

**Authors:** Yusuke Shinji, Hirotsugu Okuno, Yutaka Hirata

**Affiliations:** ^1^Department of Computer Science, Graduate School of Engineering, Chubu University, Kasugai, Japan; ^2^Faculty of Information Science and Technology, Osaka Institute of Technology, Hirakata, Japan; ^3^Department of Artificial Intelligence and Robotics, College of Engineering, Chubu University, Kasugai, Japan; ^4^Center for Mathematical Science and Artificial Intelligence, Chubu University, Kasugai, Japan; ^5^Academy of Emerging Sciences, Chubu University, Kasugai, Japan

**Keywords:** artificial cerebellum, spiking neural network, FPGA, adaptive control, motor learning

## Abstract

The cerebellum plays a central role in motor control and learning. Its neuronal network architecture, firing characteristics of component neurons, and learning rules at their synapses have been well understood in terms of anatomy and physiology. A realistic artificial cerebellum with mimetic network architecture and synaptic plasticity mechanisms may allow us to analyze cerebellar information processing in the real world by applying it to adaptive control of actual machines. Several artificial cerebellums have previously been constructed, but they require high-performance hardware to run in real-time for real-world machine control. Presently, we implemented an artificial cerebellum with the size of 10^4^ spiking neuron models on a field-programmable gate array (FPGA) which is compact, lightweight, portable, and low-power-consumption. In the implementation three novel techniques are employed: (1) 16-bit fixed-point operation and randomized rounding, (2) fully connected spike information transmission, and (3) alternative memory that uses pseudo-random number generators. We demonstrate that the FPGA artificial cerebellum runs in real-time, and its component neuron models behave as those in the corresponding artificial cerebellum configured on a personal computer in Python. We applied the FPGA artificial cerebellum to the adaptive control of a machine in the real world and demonstrated that the artificial cerebellum is capable of adaptively reducing control error after sudden load changes. This is the first implementation and demonstration of a spiking artificial cerebellum on an FPGA applicable to real-world adaptive control. The FPGA artificial cerebellum may provide neuroscientific insights into cerebellar information processing in adaptive motor control and may be applied to various neuro-devices to augment and extend human motor control capabilities.

## 1 Introduction

The human brain, a sophisticated and complex organ, possesses remarkable capabilities such as decision-making, memory formation, visual and auditory processing, language comprehension, spatial awareness, and motor control functions. These functions are thought to arise from neural networks in which a large number of neurons are diversely connected via synapses. To simulate brain function, neurons have been mathematically described using either non-spiking or spiking models. Non-spiking models are predicated on simulating the neuronal firing rate, which refers to the frequency of electrical impulses, known as spikes, emitted by neurons. This approach is based on the prevalent belief that neurons primarily encode information through variations in their firing rate. A notable example of this type of modeling is the Convolutional Neural Network, which is widely used in contemporary AI technology. However, non-spiking models are limited in their ability to accurately replicate spike-timing-dependent plasticity (STDP), an essential mechanism for learning and memory across various regions of the brain, including the visual cortex (Fu et al., 2002; Yao et al., 2004), somatosensory cortex (Allen et al., 2003; Celikel et al., 2004), hippocampus (Bi and Poo, 1998; Wittenberg and Wang, 2006), and cerebellum (Piochon et al., 2013). Therefore, to construct a neural network modeled on the brain, it is crucial to accurately simulate STDP. Spiking neuron models produce spikes to represent their activities, thereby enabling the representation of STDP. Spiking neural networks have gained attention in various fields, including those envisioning general artificial intelligence (AGI) and other specialized AI applications (Calimera et al., 2013; Pfeiffer and Pfeil, 2018; Yang and Chen, 2023; Yang et al., 2023). They have also been utilized in neuromorphic computing to emulate the structure and function of biological neural circuits efficiently (Boi et al., 2016; Osborn et al., 2018; Donati et al., 2019; Mosbacher et al., 2020). Contemporary AI development has primarily focused on mimicking the cognitive and decision-making functions of the cerebrum in humans and animals. However, the cerebellum, known for its role in motor control and learning, has also been recently identified as playing a part in higher cognitive functions and decision-making (Ito, 2008; Strick et al., 2009; Liu et al., 2022). Thus, neural networks that emulate both cerebrum and cerebellar circuits could significantly enhance the advancement of more intelligent AI and AGI. Moreover, the cerebellum has been demonstrated to be critical in the adaptive motor control of various movements, including eye movements (McLaughlin, 1967; Ito et al., 1970; Miles et al., 1986; Nagao, 1988), eye blinks (Lincoln et al., 1982; Yeo et al., 1985), arm reaching (Martin et al., 1996; Kitazawa et al., 1998), gait (Mori et al., 1999; Ichise et al., 2000), and posture (Nashner, 1976), among others (Monzée et al., 2004; Leiner, 2010). In the realm of advanced robotics, an artificial cerebellum should be highly effective for achieving human-like flexible motor control and learning. Specifically, an artificial cerebellum that operates in real-time, maintains compactness, and exhibits low power consumption, holds potential for applications in neuroprosthetics and implantable brain-machine interfaces. Such advancements may provide viable solutions to compensate for impaired motor functions.

The cerebellar neural network is well-understood regarding its anatomical connectivity and physiological neuronal characteristics, as detailed in studies by Eccles et al. (1967) and Gao et al. (2012), among many others. Since the pioneering theoretical work by Marr (1969) and Albus (1971), several artificial cerebellums have been developed. Similar to other neural network models, these artificial cerebellum models can be classified into two major types: spiking and non-spiking. A representative non-spiking cerebellar model is the cerebellar model articulation controller (CMAC), proposed by Albus (1975). CMAC has demonstrated exceptional performance and robustness as a non-model-based, nonlinear adaptive control scheme in controlling a submarine (Huang and Hira, 1992; Lin et al., 1998) and an omnidirectional mobile robot (Jiang et al., 2018). Examples of spiking models of the cerebellum include a model to explain the timing mechanism of eyeblink conditioning (Medina et al., 2000), and a model for acute vestibulo-ocular reflex motor learning (Inagaki and Hirata, 2017). Moreover, a realistic 3D cerebellar scaffold model running on pyNEST and pyNEURON simulators (Casali et al., 2019), a cerebellar model capable of real-time simulation with 100k neurons using 4 NVIDIA Tesla V100 GPUs (Kuriyama et al., 2021), and a Human-Scale Cerebellar Model composed of 68 billion spiking neurons utilizing the supercomputer K (Yamaura et al., 2020) have been constructed. A caveat with these spiking cerebellar models is their resource-intensive nature compared to non-spiking models due to the computational demands of simulating spike dynamics in neurons. Consequently, real-time simulations become challenging without substantial processing power.

In recent years, dedicated neuromorphic chips, designed for real-time computations of spiking neural networks and the simulation of various network types with synaptic plasticity, have been developed. These chips offer new possibilities for advanced neural network modeling. One such chip is the Loihi2, capable of handling up to 1 million spiking neurons across 128 cores. Each core has 192 kB of local memory, with 128 kB allocated specifically for synapses (Davies et al., 2018; Davies, 2023). This setup allows the simulation of up to 64k synapses per neuron when using 16-bit precision for synaptic weights. However, each core is designed to handle a maximum of 8,192 neurons. If a neuron requires more than 8k synapses, the number of neurons per core must be reduced. This can lead to inefficiencies, as neuron memory may remain underutilized. Another notable neuromorphic chip is the TrueNorth, which can simulate 1 million spiking neurons. However, it faces limitations with local memory for synaptic states, providing only 13 kB per core. This restricts each neuron to have just 256 synapses (Merolla et al., 2014), significantly fewer than found in cerebellar cortical neurons such as Purkinje cells. Overall, these neuromorphic chips are constrained by their memory capacity, which poses challenges for efficiently simulating complex structures like the cerebellum.

Field-programmable gate arrays (FPGAs) allow designers to program configurations of logic circuits with low power consumption, giving them an edge over central processing unit (CPUs) and graphics processing unit (GPUs) in developing specialized and efficient architectures. Several studies have implemented spiking neural networks including those of the cerebellum on FPGAs. For instance, Cassidy et al. (2011) implemented 1 million neurons on a Xilinx Virtex-6 SX475 FPGA-based neuromorphic system. Neil and Liu (2014) developed a deep belief network of 65k neurons with a power consumption of 1.5 W on an FPGA-based spiking network accelerator using a Xilinx Spartan-6 LX150 FPGA. Luo et al. (2016) implemented the cerebellar granular layer of 101k neurons on a Xilinx Virtex-7 VC707 FPGA, with a power consumption of 2.88 W. In a similar vein, Xu et al. (2018) implemented an artificial cerebellum with 10k spiking neurons on a Xilinx Kintex-7 KC705 FPGA, applying it to neuro-prosthesis in rats with an eye blink conditioning scheme. Lastly, Yang et al. (2022) implemented a cerebellar network of 3.5 million neurons on six Altera EP3SL340 FPGAs, with a power consumption of 10.6 W, and evaluated it by simulating the optokinetic response. These efforts highlight the significant role of FPGAs in neuromorphic computing, offering powerful and efficient solutions for simulating complex neural networks such as those found in the cerebellum.

One of the potential applications of the artificial cerebellum is implantable brain-machine interfaces used for neuroprosthesis. However, the chronic use of such active implanted devices raises safety concerns, particularly due to thermal effects. Studies have shown that temperature elevations greater than 3 °C above normal body temperature can induce physiological abnormalities like angiogenesis and necrosis (Seese et al., 1998), and the temperature increase due to the power consumption of an implanted microelectrode array in the brain is estimated to be 0.029°C/mW (Kim et al., 2007), suggesting that the power consumption of hardware implanted in the brain should not exceed 100 mW. From this perspective, even the FPGAs that have been used to implement cerebellar spiking neural networks (Cassidy et al., 2011; Neil and Liu, 2014; Xu et al., 2018; Yang et al., 2022), are not currently suitable for creating devices for this purpose.

In this study, we aim at constructing an artificial cerebellum that is portable, lightweight, and low power consumption. To achieve this, we utilized the Xilinx Spartan-6 and employed three novel techniques to effectively incorporate the distinctive cerebellar characteristics. First, we reduced the required number of arithmetic and storage devices while maintaining numerical accuracy. This was achieved by utilizing only 16-bit fixed-point numbers and introducing randomized rounding in the computation of numerical solutions for the differential equations that describe each spiking neuron model. Second, to facilitate the transmission of spike information between the computational units of each neuron, we installed a spike storage unit equipped with a data transmission circuit. This circuit is fully coupled between the pre- and postsynaptic neurons, effectively eliminating the latency that is dependent on the number of neurons and spikes. Third, to further decrease the number of necessary storage devices, we introduced a pseudo-random number generator. This serves as a storage device for storing information about connections between neuron models. As a result, a cerebellar cortical neural circuit model consisting of 9,504 neurons and 240,484 synapses was successfully implemented on the FPGA with low power consumption (< 0.6 W) and operation in real-time (1 ms time step). To validate the model, we compared the firing properties of a minimum scale cerebellar neuron network model on the FPGA with the same model implemented on a personal computer in Python using a 64-bit floating-point number. This comparison demonstrated that the model on the FPGA possesses sufficient computational accuracy to simulate spiking timings. Furthermore, we applied the FPGA spiking artificial cerebellum for the adaptive control of a direct current (DC) motor in a real-world setting. This demonstration showed that the artificial cerebellum is capable of adaptively controlling the DC motor, maintaining its performance even when its load undergoes sudden changes in a noisy natural environment.

## 2 Materials and methods

### 2.1 Cerebellar spiking neuronal network model

The artificial cerebellum to be implemented on the FPGA in the present study is similar to those previously constructed by referring to anatomical and physiological evidence of the cerebellar cortex (Medina et al., 2000; Inagaki and Hirata, 2017; Casali et al., 2019; Kuriyama et al., 2021). Presently, the scale of the artificial cerebellum (the number of neuron models) is set to ∼10^4^ neurons which is limited by the specification of the FPGA (XC6SLX100, Xilinx) used in the current study (see below for more detailed specs).

#### 2.1.1 Network structure

**FIGURE 1:**
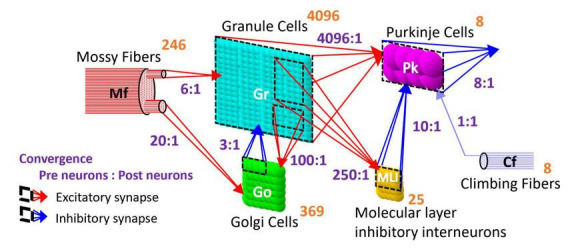
Structure of the artificial cerebellum. The numeral in orange in the top-right corner of each neuron represents the total number of neurons while the ratios indicated in blue between presynaptic and postsynaptic neurons represent the convergence ratios.

The artificial cerebellum has a bi-hemispheric structure as the real cerebellum (Figure 1). Each hemisphere consists of 246 mossy fibers (MF), 8 climbing fibers (CF), 4096 granule cells (GrC), 369 Golgi cells (GoC), 25 molecular layer inhibitory interneurons (MLI), and 8 Purkinje cells (PkC). These numbers were determined so that the model can be implemented and run in real-time on the FPGA currently employed (see below) while preserving convergence-divergence ratios of these fiber/neuron types (Figure 1, purple numbers) as close as those found in the vertebrate cerebellum (Medina et al., 2000). Note that this number of GrC (4096) has been demonstrated to be enough to control real-world objects such as a two-wheeled balancing robot robustly (i.e., independently of the initial synaptic weight values) (Pinzon-Morales and Hirata, 2015). In the cerebellar cortical neuronal network, MFs connect to GrCs and GoCs via excitatory synapses, and GrCs connect to PkCs, GoCs, and MLIs via excitatory synapses. GoCs connect back to GrCs via inhibitory synapses, while MLIs connect to PkCs via inhibitory synapses. PkCs connect to the extra cerebellar area via inhibitory synapses.

#### 2.1.2 Spiking neuron model

Neurons and input fibers in the artificial cerebellum are described by the following leaky integrate and fire model (Gerstner and Kistler, 2002; Izhikevich, 2010) described by Eqs 1–3:


(1) $$C\,\frac{\text{d}\,v\,\left(t\right)}{\text{d}\,t}=-g_{L}\,\left(v\,\left(t\right)-E_{l}\right)+i\,\left(t\right)+i_{\text{spont}}\,\left(t\right)$$

(2) $$v\,\left(t\right)=\left\{ \begin{array}{cc} v\,\left(t\right)+\left(V_{r}-V_{t\,h}\right) & \text{if}\,v\,\left(t\right)>V_{t\,h}\\ v\,\left(t\right) & \text{otherwise} \end{array}\right.$$

(3) δ⁢(t)={ 1if⁢v⁢(t)>Vt⁢h 0otherwise

where, *v*(*t*), *i*(*t*), and *i*_spont_(*t*) are the membrane potential, the input synaptic current, and the current producing spontaneous firing at time *t*, respectively. *C* is the membrane capacitance, and *g_L_* is the leak conductance. When the membrane potential *v*(*t*) exceeds the threshold *V*_*th*_, it is reset to the resting potential *V_r_*, and the unit impulse function *δ*(*t*) outputs 1. Otherwise, the unit impulse function *δ*(*t*) outputs 0. The outputs are transmitted to the postsynaptic neuron and induce postsynaptic current (see Section “2.1.3 Synapse model”). The current *i*_spont_(*t*) is to simulate the spontaneous discharge, which is generated by a uniform random number [0, 2*I*_spont_] to prevent the timing of spontaneous spikes from becoming the same between neurons. The mean spontaneous discharge current, *I*_spont_, is shown in the cerebellum model of Casali et al. (2019). The constants for each neuron/fiber type are listed in Table 1 which are the same as the previous realistic artificial cerebellums (Casali et al., 2019; Kuriyama et al., 2021) except that the parameters of the input fibers were arbitrarily defined so that their firing frequencies become physiologically appropriate.

**TABLE 1:** Neuron parameters.

Cell type	*C* [pF]	*g*_*L*_ [mS]	*E*_*L*_ [mV]	*I*_*spont*_ [pA]	*V*_*r*_ [mV]	*V*_*th*_ [mV]
MF	1.0	0.03	−70	5.0	−80	−55
CF	1.0	0.3	−70	0.015	−80	−55
GrC	3.0	1.5	−65	0.0	−75	−55
GoC	76.0	3.6	−74	36.8	−84	−42
BkC	14.6	1.0	−68	15.6	−78	−53
PkC	620.0	7.0	−62	600.0	−72	−47

#### 2.1.3 Synapse model

The synaptic transmission properties are described by the following conductance-based synapse model (Gerstner and Kistler, 2002; Izhikevich, 2010) described by Eqs 4, 5:


(4) $$\frac{\text{d}\,g_{m}\,\left(t\right)}{\text{d}\,t}=\sum_{n=0}^{N}\delta_{n}\,\left(t\right)\,w_{n+m\,N}-\frac{g_{m}\,\left(t\right)}{\tau_{s\,y\,n}}$$

(5) $$i\,\left(t\right)=-\sum_{m=0}^{M}g_{m}\,\left(t\right)\,\left(v\,\left(t\right)-E_{m}\right)$$

where, *g*_*m*_(*t*) is the synaptic conductance of the *m*-th postsynaptic neuron, δ_*n*_ (*t*) is the unit impulse of the *n*-th presynaptic neuron or fiber, and *w*_*n+mN*_ is the synaptic transmission efficiency between the *n*-th presynaptic neuron or fiber and the postsynaptic neuron. *N* is the number of presynaptic neurons. *τ*_*syn*_ is the time constant. *E_m_* is the reversal membrane potential which is positive or negative for excitatory or inhibitory synapse, respectively. As a result, the sign of the synaptic current *i*(*t*) differs between the excitatory synapse and the inhibitory synapse. *M* is the number of presynaptic neuron types. The ratios of the numbers of synaptic connections between different neuron types were as shown in Figure 1 (N_*c*1_:N_*c*2_) where N_*c*1_ and N_*c*2_ are the numbers of presynaptic and postsynaptic neurons, respectively. The connections between presynaptic and postsynaptic neurons are determined by a pseudo-random number generator described later. The initial values of synaptic transmission efficiency *w* of all synapses were assigned by Gaussian random numbers whose means and variances are different for different neuron types as listed in Table 2. In the current model, only parallel fiber (PF, the axonal extensions of GrC)–PkC synapses undergo synaptic plasticity as described below. Other synaptic efficacies were fixed at the initial value throughout the execution. The synaptic constants *τ*_*syn*_ and *E_m_* were set as shown in Table 2 based on anatomical and physiological findings (Medina et al., 2000; Kuriyama et al., 2021).

**TABLE 2:** Synapse parameters.

Postsynaptic neuron	Presynaptic neuron	Mean of initial weight *w* [pS]	Reversal potential *E* [mV]	Time constant *τ*_s*yn*_ [ms]
GrC	MF	19.5	0	0.50
GrC	GoC	3.9	−70	10.00
GoC	MF	171.9	0	0.50
GoC	GrC	31.2	0	0.50
PkC	GrC	0.0	0	0.50
PkC	BkC	3.9	−70	1.60
BkC	GrC	3.9	0	0.64

#### 2.1.4 Synaptic plasticity model

The synapses between the PF and the PkC are the loci where the memory of motor learning has been proposed to be stored (Ito, 2001; Takeuchi et al., 2008). These include long-term depression (LTD) and long-term potentiation (LTP). The present model implements plasticity described by Eqs 6, 7:


(6) $$\frac{\text{d}\,w_{\mathrm{PF}-\mathrm{PkC}}\,\left(t\right)}{\text{d}\,t}=-\gamma_{\text{LTD}}\,s_{\text{CF}}\,\left(t\right)\,q_{\text{GrC}}\,\left(t\right)+\gamma_{\text{LTP}}\,{\bar{\delta }}_{\text{CF}}\,\left(t\right)\,s_{\text{GrC}}\,\left(t\right)$$

(7) $$\tau_{\text{LTD}}\,\frac{\text{d}\,q_{\text{GrC}}\,\left(t\right)}{\text{d}\,t}=\delta_{\text{GrC}}\,\left(t\right)-q_{\text{GrC}}\,\left(t\right)$$

Here, *q*_GrC_ represents the average firing rate of the GrC, δ_GrC_(*t*) represents the unit impulse of the GrC, and ${\bar{\delta }}_{\text{CF}}\,\left(t\right)$ represents the negation of the unit impulse of the CF. δ_GrC_(*t*) turns to 1 when a spike fires, otherwise 0. The synaptic weight *w*_PF−PkC_(*t*) increases or decreases from the initial value 0 in the range of [0, 1]. When the firing of GrC and CF are synchronized, LTD occurs (Ekerot and Jömtell, 2003). This plasticity model is achieved by reducing the synaptic transmission efficiency *w*_PF−PkC_(*t*) by the product of *q*_GrC_(*t*) and the coefficient *γ*_LTD_ = 5.94 × 10^−8^ when CF spikes at time *t*. The average firing rate *q*_GrC_ is described by the low-pass filter which has the time constant (*τ*_LTD_ = 100 ms). On the other hand, LTP is induced when GrC fires and CF does not fire (Hirano, 1990; Jörntell and Hansel, 2006). This plasticity model is achieved by increasing the synaptic transmission efficiency *w*_PF−PkC_(*t*) by the product of ${\bar{\delta }}_{\text{CF}}\,\left(t\right)$, δ_GrC_(*t*), and the coefficient *γ*_LTP_ = 4.17 × 10^−7^.

### 2.2 FPGA implementation

#### 2.2.1 Design of motor control system

**FIGURE 2:**
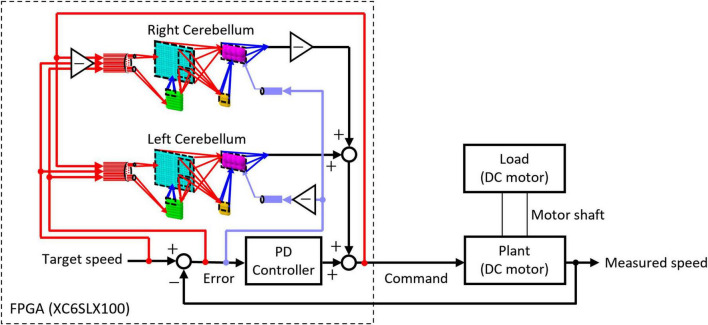
Structure of the control circuit used in the real-world experiment.

The artificial cerebellum was implemented on an FPGA (XC6SLX100-2FGG484C, Xilinx) attached to an evaluation board (XCM-018-LX100, Humandata). We used a hardware description language, VHDL to describe the artificial cerebellum which is available at [https://github.com/YusukeShinji/Shinji_Okuno_Hirata_FrontNeurosci_2024]. The left and right hemisphere models were combined as shown in Figure 2 to control real-world machines. A proportional-differential (PD) controller that simulates the pathway outside the cerebellum was implemented on the same FPGA as in the previous models (Pinzon-Morales and Hirata, 2014, 2015). The output of the cerebellum model *R*(*t*) is described by Eq. 8


(8) $$T_{P}\,\frac{\text{d}\,R\,\left(t\right)}{\text{d}\,t}=-R\,\left(t\right)+g_{P}\,\sum_{m=1}^{8}\delta_{m}\,\left(t\right)$$

where δ_*m*_(*t*) is the unit impulse representing the spiking output of the *m*th PkC, *g*_*P*_ = 0.35 is a gain coefficient, and *T*_*P*_ = 310 ms is the time constant of a low-pass filter that describes the relationship between *R*(*t*) and *δ*_*m*_(*t*). The cerebellum model outputs *R*_*L*_(*t*) and *R*_*R*_(*t*) of the left and right hemispheres are linearly summed with the output of the PD controller to obtain the command value *y*(*t*) of DC motor as described in Eq. 9 where proportional and derivative parameters of the PD controller multiplied by the error signal *E*(*t*) are *G*_*P*_ = 0.00635 and *G*_*D*_ = 0.00001, respectively.


(9) $$y\,\left(t\right)=G_{P}\,E\,\left(t\right)+G_{D}\,\left(E\,\left(t\right)-E\,\left(t-\Delta \,t\right)\right)+R_{L}\,\left(t\right)-R_{R}\,\left(t\right)$$

The Command value *y*(*t*) is converted into a pulse-width modulated (PWM) voltage signal and then fed to the motor to be controlled. The control object currently tested is a DC motor (JGA25-370, Open Impulse). As a load was added to the control object, the same type of DC motor was connected co-axially. The load was imposed by short-circuiting the DC motor via a relay circuit controlled by the same FPGA. The produced motion of the control object in response to a given target speed was measured by an encoder and fed back through a hole sensor to calculate the error. The error is sent to the artificial cerebellum as CF activity which induces PF–PkC synaptic plasticity (see Section “2.1.4 Synaptic plasticity”). Other input modalities to the artificial cerebellum via MFs are target speed, error, and the copy of motor command (efference copy) as in the real oculomotor control system (Noda, 1986; Hirata and Highstein, 2001; Blazquez et al., 2003; Huang et al., 2013).

#### 2.2.2 Design of computation, communication, and memory systems

**FIGURE 3:**
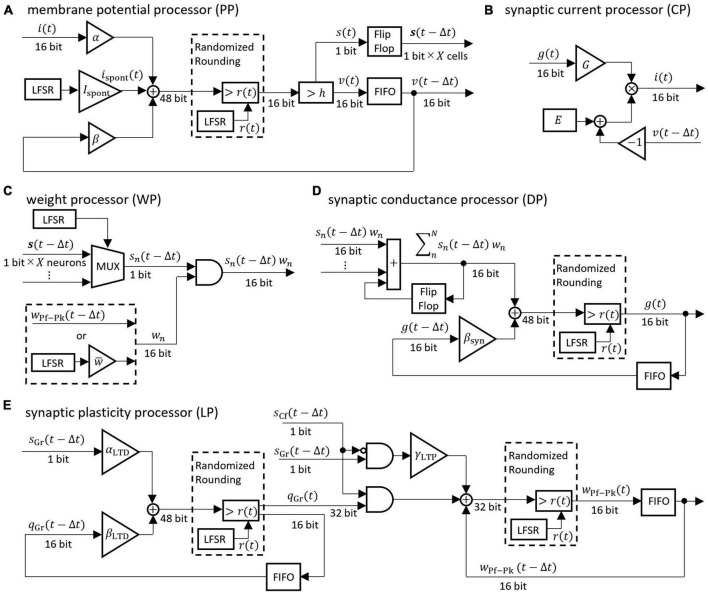
Block diagrams of the neural processor. **(A–E)** Processing devices for membrane potential, synaptic current, synaptic weight, synaptic conductance, and synaptic plasticity, respectively. The coefficients are defined as follows: *α* = 1/*C*, *β* = 1 − *g*_*L*_/*C*, *β*_syn_ = 1 − 1/*τ*_syn_, *α*_LTD_ = *γ*_LTD_/*τ*_LTD_, *β*_LTD_ = 1 − 1/*τ*_LTD_. For the synaptic transmission efficiency *w* in **(C)**, only the PF-PkC synapse uses the numerical value obtained by calculating LTD and LTP according to **(E)**. Adder and AND gates are composed of logic circuits in the look-up table (LUT). The DSP slice in the FPGA was used for the triangular block representing the gain and the multiplication. The LFSR is a linear feedback shift register, each of which outputs a random number *r*(*t*) that differs depending on the initial seed. The flip-flop is a storage element that is constructed by registers contained in the LUT in the FPGA. The FIFO is a storage element that uses the FPGA’s block RAM in a first-in-first-out format. The multiplexer (MUX) is a data selector. In **(C)**, the MUX selects the impulse of one neuron from the flip-flop storing impulses of *X* neurons. In the **(A,D,E)**, randomized rounding is a rounding element that rounds up if the random number generated by LFSR is smaller than the fraction bits and rounds down otherwise.

The differential equations describing the neuron model were discretized by the Euler method and implemented as digital circuits shown in Figure 3. Digital circuits that simulate neuron groups in the model are interconnected to form the cerebellar neural network shown in Figure 1. Figure 3A is the membrane potential processor (PP), B is the synaptic current processor (CP), C is the weight processor (WP), D is the synaptic conductance processor (DP), and E is the synaptic plasticity processor (LP).

**FIGURE 4:**
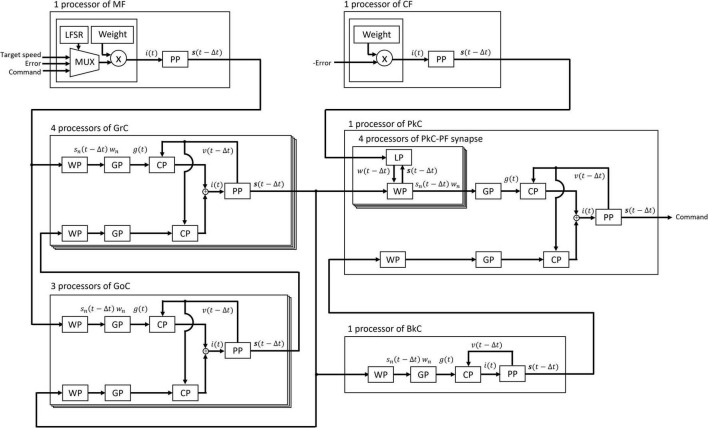
Processing circuit of the artificial cerebellum. Overlapping frames indicate parallel processing.

These processors adopted two parallel processing methods to complete the processing within a time step (1 ms). The first parallel processing method is pipeline processing used in all the processors in Figure 3. For example, pipeline processing in WP reduces the execution time by processing the second synapse at the MUX simultaneously with the stage of processing the first synapse at the AND. The second parallel processing method is the parallel operation of the dedicated processors described above. As shown in Figure 4, a number of PP, CP, DP, WP, and LP are provided for parallel processing. Four processors are provided to process GrCs in parallel due to their large number of neurons. To process PkCs, 4 WPs, and 4 LPs are provided because PkC has a large number of synapses despite a small number of neurons (8 neurons). Three processors are provided to complete GoC processing during the PkC processing. One processor is provided to complete BkC processing during PkC processing.

In addition to these parallel processing, we adopted the following three new methods for implementing the artificial cerebellum efficiently on the FPGA within the limits of the number of logic blocks and time steps required for actual motor control.

##### 2.2.2.1 Fixed-point arithmetic and randomized rounding

In order to reduce computational cost and memory usage, 16-bit fixed-point numbers were adopted instead of floating-point numbers in the FPGA. However, round-off errors that occur in fixed-point arithmetic can degrade the precision of computation. In order to minimize the accumulation of rounding errors, randomized rounding was adopted. This method compares a fraction that is supposed to be rounded up or off with random numbers. Namely, if the fraction is less than the random number, it is rounded up while if the fraction is greater than or equal to the random number, it is truncated. The average rounding of random numbers is distributed around the fractions and can be rounded unbiasedly. We employed uniform random numbers generated by a linear feedback shift register (LFSR). Although an LFSR is a pseudo-random number generator with periodicity, the bit width of the LFSR used in this study (32-bit) provides a sufficiently long period and can keep the bias in randomized rounding small.

##### 2.2.2.2 Fully coupled spike transfer circuit

**FIGURE 5:**
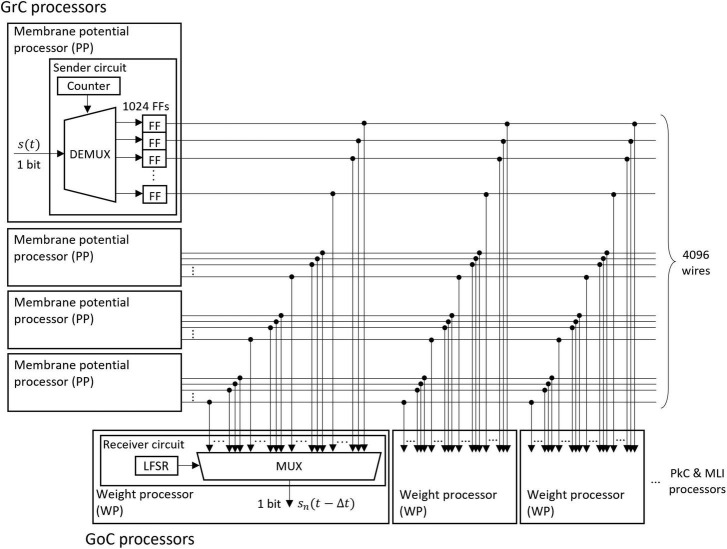
Structure of the fully coupled spike transfer circuit. The unit impulse δ(*t*) calculated by the membrane potential processor (PP) is stored in the flip-flop (FF). The outputs of all FFs of the presynaptic neurons are connected to the multiplexer (MUX) of all weight processors (WP) of the postsynaptic neuron. By receiving the output of the linear feedback shift register (LFSR), the MUX randomly selects a presynaptic neuron and outputs the impulse.

The memory of the unit impulse *δ*(*t*) is frequently referenced by many processors. Short memory latency is required to take advantage of parallel processing. Therefore, we designed a parallel I/O interface composed of sender circuits and receiver circuits. We used this interface for the transmission of impulses between all neurons. An example of the interface is the transmission circuit from GrC to GoC as shown in Figure 5. The interface consists of sender circuits in the processor of GrC, which is the presynaptic neuron, and receiver circuits in the processor of GoC, which is the postsynaptic neuron.

A sender circuit is composed of a counter, a demultiplexer (DEMUX), and flip-flops (FFs). The number of FFs corresponds to the number of model neurons simulated in the PP of GrC; the number is 1024 in this case. When the membrane potential processing ends and the impulse is generated in the PP, the DEMUX receives the impulse and stores the event in one of the FFs depending on the counter output, which represents the neuron identifier (ID) of the impulse. The total number of impulses handled by this interface is 4096 because four sender circuits work in parallel. Here, the ON/OFF state of one wire of the FF output represents an impulse (whether spiked or not) of one neuron in a certain 1 ms.

A receiver circuit is composed of an LFSR and a multiplexer (MUX), which selects one signal from its 4096 input signals depending on the LFSR output. The LFSR outputs pseudo-random numbers, each of which represents the ID of the presynaptic neuron connected to the postsynaptic neuron processed at the period. The output of the LFSR is updated every 1 clock. One of the GoC (postsynaptic neuron) processors simulates 123 GoCs and three GoC processors simulate 369 GoCs in total. Each GoC is connected to 100 presynaptic GrC neurons, and the IDs of the connected GrC neurons are determined by the LFSR. Each receiver circuit repeats the spike read procedure 100 × 123 times. When the circuit completes one cycle of the procedure, the LFSR is reset to the initial value which varies for each receiver circuit, and the impulse from the same presynaptic neuron ID is read in the next time step. By branching receiver circuits, the reading procedure of impulses is processed in parallel.

##### 2.2.2.3 Pseudo-random number generator to represent neural connections

In order to reduce memory usage, we adopted a pseudo-random number generator in place of a memory that stores information on neural connections. When implementing the artificial cerebellum on an FPGA, it is necessary to store the neuron ID of presynaptic and postsynaptic neurons. As shown in Figure 3C, the ID is used by MUX to output impulses of a desired neuron group. The memory capacity required for storing the ID is the product of the number of pre-synaptic neurons, the number of post-synaptic neurons, and the convergence rate, which is huge (approx. 40 million bytes). However, the capacity of the internal RAM, which is the RAM embedded in an FPGA and provides a much wider bandwidth than the external RAM, is very limited. The effective use of the internal RAM is the key factor for implementing many neurons in an FPGA. A large amount of internal RAM should be used for storing differential equation variables, not for the ID of neural connections. Because the neural connection in our model is defined by random numbers that are unchanged through an operation, we used an LFSR to achieve uniform random numbers that define neural connections. Since an LFSR is composed only of XORs and FFs, internal RAMs are unnecessary. Similarly, synaptic weights that don’t have synaptic plasticity were generated by the LFSR.

## 3 Results

### 3.1 Specifications

The neural network constituting the artificial cerebellum contains 9,504 neurons (including MFs and CFs) with 240,484 synapses. The hardware resources used for the artificial cerebellum construction are shown in Table 3. Block memory and Multiplier are internal RAM and DSP slices in the Spartan-6 FPGA chip. Distributed memory is RAM that uses look-up tables. At the FPGA clock frequency of 40 MHz, the calculation time for each hemisphere of the artificial cerebellum is 0.40 ms. The entire control circuit, including both hemispheres, completes all the computation in 1 ms time interval. Considering that the maximum firing rate of neurons in the cerebellum is about 500 spikes/s (Ito, 2012), 1 ms is fast enough for simulating the cerebellum. Therefore, real-time operation of the artificial cerebellum with this configuration is possible. The maximum power consumption at a clock frequency of 40 MHz and a device temperature of 25°C was estimated to be 0.6 W by the Xilinx Power Estimator.

**TABLE 3:** Specifications of the artificial cerebellum.

Timing issues							
Clock frequency					40 MHz		
Time step					1 ms		
**Estimated power consumption in 25°C**							
Maximum power consumption					0.6 W		
Typical power consumption					0.4 W		
**Primitive statistics**							
	**Membrane potential processors**	**Synaptic current processors**	**Weight processors**	**Synaptic conductance processors**	**Synaptic plasticity processors**	**Others**	**Total**
Look-up table	22,500	1,318	20,721	3,904	3,972	4,561	56,976
Flip-flop/latch	11,118	556	3,667	2,554	1,572	3,017	22,484
Block memory	22	0	65	98	0	70	255
Distributed memory	0	0	0	272	197	153	622
Multiplier	22	17	90	17	8	6	160

### 3.2 Simulation to evaluate the randomized rounding

To evaluate the effect of rounding error, we calculated one neuron model with the following three methods: (1) Python with 64-bit floating-point number and half-up rounding, (2) Xilinx ISE Simulator and 16-bit fixed-point number and half-up rounding, (3) Xilinx ISE Simulator with 16-bit fixed-point number and randomized rounding. As a simulation with high calculation accuracy, we used Python’s float type which is a 64-bit floating-point number and performed the simulation on a personal computer.

We simulated one GrC receiving inputs from one GoC and one MF. The input signals sent from GoC and the MF are impulses whose firing time is determined by uniform random numbers generated by an LFSR. The simulation was performed under the condition that the GoC fires at an average of 31 spikes/s and the MF fires at an average of 62 spikes/s so that the effects of rounding error can be easily evaluated. In this simulation, the weight between GrC and GoC was set to 93.8 pS, and that between GrC and MF was set to 320.0 pS.

**FIGURE 6:**
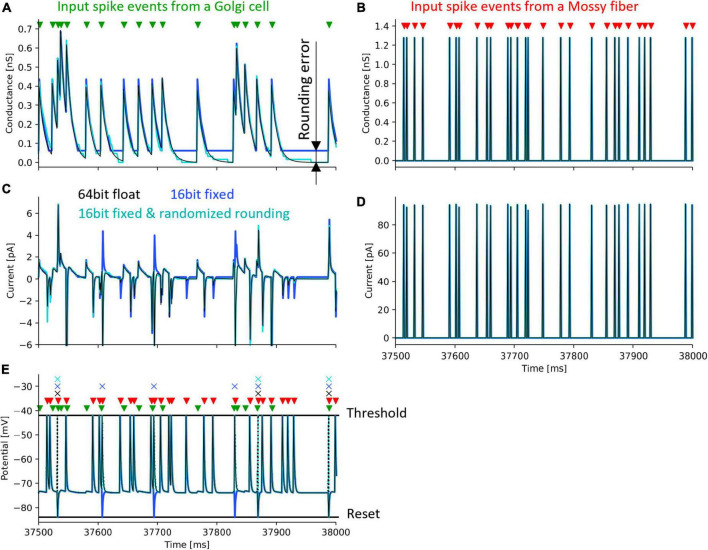
Simulation of a granule cell to verify the accuracy of randomized rounding. Black lines and cross marks depict a simulation result using a 64-bit floating-point number and half-up rounding, calculated in Python. Blue lines and cross marks represent a simulation result using a 16-bit fixed-point number and half-up rounding calculated in the Xilinx ISE Simulator. Cyan lines and cross marks show a simulation result using a 16-bit fixed-point number and randomized rounding calculated in the Xilinx ISE Simulator. Green and red triangles denote the spike timing of input to a GrC from a GoC and an MF. Cross marks illustrate the spike timing of the output of the GrC. **(A)** postsynaptic conductance between the GrC and the GoC. **(B)** postsynaptic conductance between the GrC and the MF. **(C)** postsynaptic current between the GrC and the GoC. **(D)** postsynaptic current between the GrC and the MF. **(E)** membrane potential of the GrC. Dotted lines represent changes in membrane potential during spikes which were not stored in the FPGA.

Figure 6 shows the simulation results. The black lines in the figure plot the results simulated with a 64-bit floating-point number. Simulations with 16-bit fixed-point numbers cause rounding errors when computing the differential equations of synaptic conductance and membrane potential due to the limited number of digits. As a result, the synaptic conductance computed with 16-bit fixed-point numbers and half-up rounding does not converge to 0 nS (Figure 6A, blue line), resulting in the positively biased membrane potential, and the increased spike frequency (Figures 6C, E, blue line and cross mark, respectively). In contrast, using randomized rounding, the synaptic conductance converges to 0 nS (Figure 6A, light cyan line), the membrane potential is not biased, and the frequency of spike occurrence is almost the same as that computed with 64-bit floating-point numbers (Figures 6C, E, light cyan line and cross mark). The synaptic conductance computed with a 64-bit floating-point and that computed with a 16-bit fixed-point number and randomized rounding are approximately equal. Even if an error occurs in the computation of synaptic conductance, the conductance computed with a 16-bit fixed-point number and randomized rounding converges to 0 nS while no spike comes to the neuron, so the accumulated error can be canceled. In the simulation computed with 64-bit floating-point numbers, the mean firing rate of GrCs during 50 s was 6.58 spikes/s. The mean firing rate difference between the simulation with 64-bit floating-point numbers and that with 16-bit fixed-point numbers was 1.64 spikes/s. The mean firing rate difference between the simulation with 64-bit floating-point numbers and that with 16-bit fixed-point numbers and randomized rounding was 0.030 spikes/s. These results assure that the calculation accuracy can be maintained by using randomized rounding when computing differential equations that describe a neuron model with fixed-point numbers.

### 3.3 Real-world adaptive machine control

In order to evaluate the capability of the artificial cerebellum in adaptive actuator control in a real-world environment, we employed a DC motor and imposed a load that varies in intensity over time. As shown in Figure 2, the FPGA controls the DC motor via an inverter in the control circuit. The rotation speed of the controlled object was fed back to the FPGA by the Hall effect sensor. The rotation speed error, which is calculated by subtracting the measured speed from the target speed, was input to the MFs and CFs of the artificial cerebellum.

Another DC motor was connected to the shaft of the controlled motor to impose a load. During the experiment, the switch in the circuit was opened to make the Load-OFF state and was closed to make the Load-On state. The resistance of the switch was 13.5Ω. The switch was opened and closed by the signal sent from the FPGA. The target time course of the rotation speed was a sine wave with an amplitude of 32 rotations per second (rps) and a period of 2.048 s. The load was turned on in the 120th cycle, turned off in the 240th cycle, and turned on again in the 300th cycle, at which the control of the artificial cerebellum had been stable.

**FIGURE 7:**
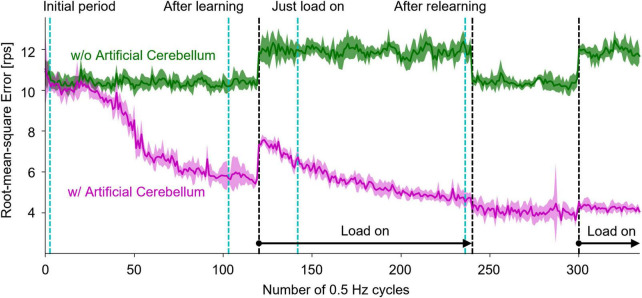
Results of real-world motor control with and without the artificial cerebellum. The magenta and green lines represent the results with and without the artificial cerebellum, respectively. The solid line and the shaded area indicate the mean and plus-minus 1 standard deviation, respectively, over 10 experiments. The black dashed lines indicate the periods where the load was imposed. The cyan dashed lines denote the periods evaluated in Figure 8.

Figure 7 shows the results of motor control experiments repeated 10 times each with or without the artificial cerebellum. Because the initial weights of the synapses between PFs and PkCs were set to 0, the amount of error was the same with and without the artificial cerebellum at the beginning of the experiment. With the artificial cerebellum, the amount of error started from the same level as the PD controller alone. The adaptation did not start immediately due to the influence of noise in the real-world environment. The error started to decrease from around the 50th cycle due to motor learning in the artificial cerebellum. When a load was imposed in the 120th cycle, the error rose sharply but gradually decreased as the artificial cerebellum adapted to the load. By contrast, the error increased with a much smaller amount at the timing of Load-Off in the 240th cycle and Load-On in the 300th cycle, demonstrating generalization in adaptation to both Load-Off and Load-On states.

**FIGURE 8:**
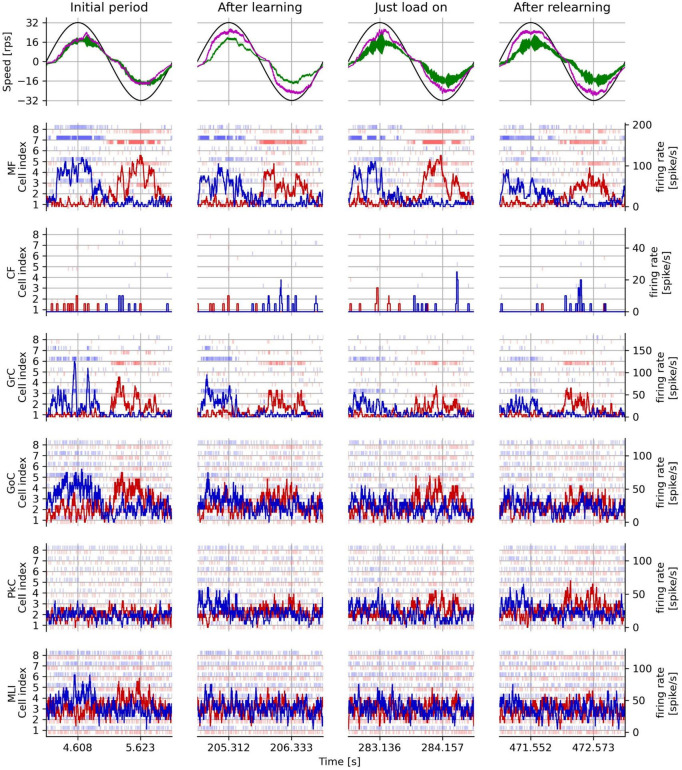
Measured rotation speed of the plant and activities of each neuron type in the real-world adaptive motor control experiment. The left column shows the initial period, the period after learning, the period after load application, and the period after relearning with load on in one trial. The first row from the top shows the target speed (black line), the measured speed without the artificial cerebellum (green line), and the measured speed with the artificial cerebellum (magenta line). The second and subsequent rows from the top show the responses of each type of neuron in the left cerebellum (blue) and the right (red) hemisphere of the artificial cerebellum. The light vertical line markers indicate the spike timing of 8 representative neurons, while and the dark solid lines indicate their average firing rates.

Figure 8 shows the rotation speed of the controlled DC motor and the activities of representative neuron models in the following four periods: initial period, after learning, just load-on, and after relearning corresponding to each triangle marked on the horizontal axis in Figure 7. In the top panels of Figure 8, the black, green, and magenta lines plot the target speed, the measured speed controlled without the artificial cerebellum, and the measured speed controlled with the artificial cerebellum, respectively. In the initial period (left top panel), the measured speed controlled with the artificial cerebellum (magenta) was smaller than and delayed to the target speed (black). This situation was similar to the speed controlled without the artificial cerebellum (green). After learning (2nd column from the left, top panel), the measured speed controlled with artificial cerebellum (magenta) improved the amplitude and the delay. After load on (2nd to the right column, top panel), the speed controlled with artificial cerebellum (magenta) decelerated just before it reached the maximum speed. After relearning (right top panel), the control with artificial cerebellum (magenta) improved the deceleration just before it reached the maximum speed, and further improved the delay after switching the direction of rotation. The raster plots in the 2nd to the bottom panels of Figure 8 show spike timings of 8 neurons randomly selected from each neuron type in the left (red) and right (blue) cerebellar hemispheres. The red and blue lines in these panels are the average firing rate of the 8 neurons in the left and right cerebellar hemispheres, respectively. MF and CF firing rates (2nd and 3rd row, respectively) showed almost the same responses in all cycles. The firing rate of GrC (3rd row from the top) decreased significantly in the after-learning period compared to those in the initial period. The same trend is observed in the relationship between GrC firing rates in the after-relearning period and those in the just-load-on period. The firing rates of GoC (3rd row from the bottom) and MLI (1st row from the bottom) showed similar changes to those of GrC. Because the initial weights of the synapse between PF and PkC were 0, the firing rate of PkC (2nd row from the bottom) showed only a spontaneous firing rate in the initial period. In the after-learning period, the firing rate of PkC increased mainly after switching the direction of rotation. In the just-load-on period, the firing rate of PkC was active and was similar to that in the after-learning period. In the after-relearning period, the firing rate of PkC was further increased after switching the direction of rotation. These results assure that the FPGA artificial cerebellum may provide useful information as to signal processing executed in the cerebellar cortical neural network consisting of these neuron types connected in a manner unique to the cerebellum while working as an adaptive motor controller in the real world.

## 4 Discussion

We implemented for the first time a spiking artificial cerebellum on an FPGA, which accurately replicated the structure of cerebellar neural circuitry, including MLIs, and runs in real-time. We have demonstrated that the spiking neuron model on the FPGA has sufficient computational accuracy to simulate spiking timings, as shown in Figure 4. Furthermore, we have shown its capability as a real-time adaptive controller in real-world experiments, as shown in Figures 5, 6. In the following, we discuss briefly the general advantages of the current implementation of an artificial cerebellum, as well as the three key techniques used in the current FPGA implementation of the artificial cerebellum. Lastly, we discuss potential neuroscientific insights gained from this study.

### 4.1 Advantages of the current implementation

Neuromorphic chips have been shown to support neural networks with lower power consumption compared to CPUs and GPUs. IBM’s TrueNorth integrates 1 million neurons onto a single chip and can support neural network inference workloads with a low power consumption of 70 mW (Merolla et al., 2014). Loihi integrates 128,000 neurons onto a single chip and can support application demonstrations such as adaptive robot arm control and visual-tactile sensory perception with less than 1 W (Davies et al., 2021). Loihi 2 integrates 1 million neurons onto a single chip and can support the PilotNet SDNN for signal processing tasks with approximately 74 mW (Shrestha et al., 2024). However, as outlined in the introduction, these chips may face limitations in simulating neurons that form extensive synaptic connections, exemplified by cerebellar PkCs, which establish approximately 200,000 PF connections each. Consequently, the development of an artificial cerebellum with these contemporary neuromorphic chips remains unachieved, presenting challenges in directly comparing the power efficiency with our FPGA-based artificial cerebellum.

In previous studies (Cassidy et al., 2011; Neil and Liu, 2014; Luo et al., 2016; Xu et al., 2018; Yang et al., 2022), FPGAs have been used to build spiking cerebellar models that efficiently compute spiking neural networks. However, these spiking cerebellar models are not suited for implantable brain-machine interfaces due to their high power consumption, which results from the use of high clock rates or multiple chips. In Table 4, we present the FPGA implementation of the artificial cerebellum used in this study, along with a comparison to previous devices. In this study, an artificial cerebellum was constructed with a network sufficient to have learning capabilities with lower power consumption than previous devices, using a single FPGA chip.

**TABLE 4:** Comparison with previous spiking neural networks on the FPGA.

References	Device	Model	Power [W]	Time step [ms]	Clock [MHz]	Total neurons	Total synapses	Test task of learning
Cassidy et al., 2011	Xilinx Virtex-6 SX475 one chip	LIF network	None	1	200	1 M	1024 k	None
Neil and Liu, 2014	Xilinx Spartan-6 LX150 one chip &128MB DDR2 RAM	Spiking deep belief network	1.5	152	400	65 k	647 k	MNIST
Luo et al., 2016	Xilinx Virtex-7 VC707 one chip	Spiking cerebellar granular layer	2.88	1	122	101 k	900 k	None
Xu et al., 2018	Xilinx Kintex-7 KC705 one chip	Spiking cerebellar network	None	1	31	10 k	146 k	Eyeblink conditioning *in vivo*
Yang et al., 2022	Altera Stratix III EP3SL340 six chips	Spiking cerebellar network	10.58	0.2	50	3.5 M	218 M	Optokinetic response adaptation
This study	Xilinx Spartan-6 LX100 one chip	Spiking cerebellar network	0.6	1	40	9.5 k	240 k	DC motor adaptive control

Furthermore, FPGA design with VHDL can also be used for ASIC implementation, which can eliminate unused elements and redundant wiring. It has been shown that ASIC can reduce semiconductor area to 1/21, delay to 1/2.1, and power consumption to 1/9.0 compared to FPGA in a 90 nm process (Kuon and Rose, 2007). Although the FPGA used in the current study was a spartan-6 with a 45 nm process and cannot be directly compared, it may be possible to achieve 65 mW when implemented on an ASIC. This is below the brain power requirement of 100 mW, which can cause thermal damage (Kim et al., 2007).

### 4.2 Three techniques to implement the artificial cerebellum on the FPGA

#### 4.2.1 Fixed-point arithmetic and randomized rounding

Computing numerical solutions of differential equations in fixed-point numbers produces a constant rounding error unless randomized rounding is used (Figure 4). This type of rounding error is commonly encountered in first-order lag differential equations describing the synapse model and the neuron model. The first-order lag differential equation is expressed by Eq. 10:


(10) $$\tau \,\frac{d\,y\,\left(t\right)}{d\,t}=u\,\left(t\right)-y\,\left(t\right)$$

where *u*(*t*) is the input, *y*(*t*) is the output, and *τ* is the time constant. The formula for rounding half up the binary number of arbitrary digits *n* of the first-order lag differential equation discretized by the Euler method can be expressed by Eq. 11:


(11) $$y\,\left(t+\Delta \,t\right)=\left\lfloor \frac{\frac{\Delta \,t}{\tau }\,u\,\left(t\right)+\left(1-\frac{\Delta \,t}{\tau }\right)\,y\,\left(t\right)}{2^{-n}}+2^{-1}\right\rfloor \,2^{-n}$$

where ⌊*x*⌋ is the floor function. *n* is the number of digits representing the fractional part of *y*(*t*). When the input *u*(*t*) is 0 and the output *y*(*t*) is smaller than the following particular value, *y*(*t*) ceases to change due to a constant rounding error. This condition is −2^−*n*−1^ *τ*/Δ*t* ≤ *y*(*t*) < 2^−*n*−1^ *τ*/Δ*t*. This means that the longer the time constant (the closer the coefficient *τ* is to 1), the larger the range of rounding errors. For example, let the coefficient *τ*/Δ*t* be 2^5^ and fixed-point numbers of (1−Δ*t*/*τ*) and *y*(*t*) have the 16-bit fractional part. The fractional part of the multiplying (1−Δ*t*/*τ*) and *y*(*t*) is 32 bits, so it must be rounded to 16 bits (= *n*). At this time, the term that the constant rounding error remains is −2^−12^ ≤ *y*(*t*) < 2^−12^, which is within 16 bits of significant digits of *y*(*t*). In this way, if the output *y*(*t*) matches the condition described above, it is necessary to perform randomized rounding. In particular, when computing second-order integration in the synapse model and the neuron model, randomized rounding should be adopted because the bias is caused by the constant rounding error in the first step and the constant rounding error accumulates in the second step.

Thus far, 16-bit fixed-point numbers and randomized rounding have been adopted to streamline deep learning operations, and have shown accuracy comparable to 32-bit floating-point numbers (Gupta et al., 2015). In FPGA spiking artificial cerebellum, we showed that 16-bit fixed-point numbers and randomized rounding can compute spikes with comparable accuracy to 64-bit floating-point numbers. Hence, fixed-point arithmetic and randomized rounding are capable of efficient implementations while preserving arithmetic accuracy. When calculating a differential equation with an extended time constant and minimal changes per time step, insufficient bit length in fixed-point numbers can pose a problem. The changes might occur outside the range accommodated by the given bit length. Consequently, even with fixed-point arithmetic that employs randomized rounding, these slow changes cannot be accurately stored in memory. This limitation results in significant rounding errors, particularly in scenarios involving gradual alterations. Nonetheless in the cerebellar neuronal network model currently implemented, the accuracy and effectiveness of learning, potentially impacted by our implementation of a 16-bit fixed-point number and randomized rounding, should remain largely unaffected because the time constants of the neuron models are small enough. This assertion is supported by the data presented in Figure 6E, which demonstrates that the spike timings and firing rates of GrCs and other neuron models are highly comparable to those simulated using 64-bit floating-point numbers. Given that the neural basis of cerebellar motor learning is determined by the combined spike timings and firing rates of GrCs (activity of PFs) and CFs, the impact of employing a 16-bit fixed-point number and randomized rounding on the learning’s accuracy and effectiveness is considered negligible.

#### 4.2.2 Fully coupled spike transfer circuit

In order to achieve efficient communication of impulses, we designed a fully coupled spike transfer circuit, which is a parallel input/output interface. The strengths of using this interface for FPGA implementation of the artificial cerebellum include the following points.

First, it reduces the time required for spike transfer. Since the GrC in the cerebellum has PFs connecting to a large number and variety of neurons over a wide area, memories of the impulse are accessed at a very high frequency. In our artificial cerebellum, 8 WPs simultaneously access the impulse memories at 40 MHz. If the event is stored in a memory that is accessible only in serial, the time required to write/read the impulse increases as a multiplier of the number of presynaptic and postsynaptic neuron processors. In this interface, All the impulses are accessible in parallel from the processors that receive the impulses because all the impulses are stored in the FFs whose outputs are accessible from any circuits in the FPGA. In addition, each read-and-write procedure completes in 1 clock, even if the number of neurons is large and there are many processing circuits for writing and reading. This interface reduces the spike transfer time drastically.

Second, the spiking neuron model and the FPGA are suitable for implementing the fully coupled spike transfer circuit. In this interface, the numbers of the wires and the input ports of the MUX increase as the number of neurons increases. However, the increase in the circuit scale can be kept small because information transferred in this interface is the impulse, which can be expressed with only 1 bit. On the other hand, FPGA essentially has many lookup tables including logic circuits and many wires (the FPGA used in this study has 63,288 lookup tables with 380,000 wires). Therefore, the interface for a larger number of presynaptic/postsynaptic neurons is installable in an FPGA.

When scaling up to implement a larger network, the Multiplexer (MUX) circuit, responsible for reading impulse data from the Flip-flop, tends to become excessively large. This increase in size can lead to a delay exceeding one clock cycle. In such instances, it may be necessary to introduce a delay in subsequent processes to compensate for the MUX-induced delay.

#### 4.2.3 Pseudo-random number generator to represent neural connections

As previously stated in Section “2.2.2.3 Pseudo-random number generator to represent neural connections,” the utilization of an LFSR as a pseudo-random number generator serves to conserve memory. However, it should be noted that these pseudo-random numbers can have an impact on the structure of the cerebellar network.

The convergence was established as a fixed-point number of mean values obtained through autopsies. However, due to the uniform distribution and overlap of the pseudo-random numbers determining which presynaptic neuron to connect to, it is possible for the same presynaptic neuron to be selected several times. As the processing of synapse formation is equal when the same presynaptic neuron is chosen, this is equivalent to doubling the weight and decreasing the convergence by one. When the weight is doubled, the initial value is determined by a random number, leading to potential bias in rare cases.

Divergence is the sum of some uniformly distributed pseudo-random numbers. The range of these numbers is [*a*, *b*] = [1, number of presynaptic neurons− 1], with a mean of μ = (*a* + *b*)/2 and a variance of σ = (*b* − *a*)^2^/12. When the number of uniform random numbers (= *n*) is sufficiently large, the central limit theorem states that divergence follows a normal distribution with parameters *N*(*nμ*, *nσ*/12) and lies within the range [*an*, *bn*]. However, it should be noted that the convergence and divergence present in the cerebellum, as observed through anatomy, are not constant (Eccles et al., 1967; Ito, 2012), hence the utilization of pseudo-random numbers in this model. Additionally, as each PkC receives connections from more than 100,000 GrCs, this model does not use random numbers and instead assumes projections from all GrCs.

Furthermore, each MF branches to form 20–30 presynaptic sites and inputs to various GrCs and GoCs (Eccles et al., 1967). The axons of GrCs project as PFs over a distance of approximately 2 mm in the cerebellum (Eccles et al., 1967), while the maximum linear extent of GoC axons is 650 ± 179 μm in the sagittal plane and 180 ± 40 micrometers in the medial-lateral plane (Barmack and Yakhnitsa, 2008). Astrocyte axons in the molecular layer of the cerebellum are approximately 200 micrometers in diameter (Barmack and Yakhnitsa, 2008). Based on a reported density of GrCs of 4 × 10^6^ mm^3^ (Solinas et al., 2010), the granular layer of this model, containing 4096 GrCs per hemisphere, corresponds to a cubic volume of 100 micrometers on each side. In the model, each neuron’s axon is sufficiently long to span the entire volume of the artificial cerebellum, allowing for the selection of neuronal connections among all neurons. However, it is important to note that these neuronal connections are abstracted and described as random in the model.

### 4.3 Neuroscientific insights

To comprehend the neuronal circuitry of the cerebellum, it is necessary to record neuronal activity and synaptic transmission efficiency. However, due to the high density and large number of neurons in the cerebellum, measuring neuronal activity and synaptic transmission efficiency comprehensively is challenging, even with methods such as calcium imaging. In contrast, real-time simulation of an artificial cerebellum using an FPGA, coupled with the capability to record and communicate neural activity, enables the detailed logging of both neural activity and synaptic weights within the cerebellum. Moreover, the FPGA-implemented artificial cerebellum is capable of simulating the plasticity of multiple PF-PkC interactions in real-world scenarios. Future applications involving direct control of actual devices will offer valuable insights into the regulation of synaptic weights by long-term depression (LTD) and long-term potentiation (LTP). This research holds considerable potential implications for the field of neuroscience.

## 5 Summary and conclusion

We have proposed an architecture designed to efficiently simulate a key feature of the cerebellum: the presence of a large number of synapses per neuron. This feature is typically challenging to simulate with general-purpose spiking neural network processors. By implementing this architecture on a widely-used FPGA, we have created an artificial cerebellum comprising left and right hemispheres, which includes 9,504 neurons and 240,484 synapses. This model incorporates major cerebellar cortical neuron types and their synaptic connections. Additionally, it is characterized by low power consumption (less than 0.6 W) and operates in real-time with a 1 ms time step. We have successfully verified the operation of the artificial cerebellum, demonstrating that it learns correctly in real-world conditions. This compact and low-power artificial cerebellum could be inserted into the brain with minimal additional effort and applied to neuroprosthesis as implementable brain-machine interfaces to restore and enhance cerebellar functions in the future.

## Data availability statement

The raw data supporting the conclusions of this article will be made available by the authors, without undue reservation.

## Author contributions

YS and YH designed the study, the main conceptual ideas, and the proof outline. YS developed the software and collected the data. HO provided a part of the software, YS wrote the manuscript, HO and YH edited the manuscript. All authors contributed to the article and approved the submitted version.
